# Cross-sectional analysis of nutrition and serum uric acid in two Caucasian cohorts: the AusDiab Study and the Tromsø study

**DOI:** 10.1186/s12937-015-0032-1

**Published:** 2015-05-14

**Authors:** Svetlana N Zykova, Hilde M Storhaug, Ingrid Toft, Steven J Chadban, Trond G Jenssen, Sarah L White

**Affiliations:** 1 Clinical Research Department, University Hospital of North Norway, 9038 Tromsø, Norway; 2 University of Tromsø-The Arctic University of Norway, Tromsø, Norway; 3 Royal Prince Alfred Hospital, Sydney, Australia; 4 Sydney Medical School, University of Sydney, Sydney, Australia; 5 Oslo University Hospital, Oslo, Norway

**Keywords:** Uric acid, Micronutrients, Macronutrients, Food categories, Vitamins, Diet, The Tromsø study, Ausdiab

## Abstract

**Background:**

Hyperuricemia can lead to gout, and may be a risk factor for cardiovascular events, hypertension, diabetes and renal disease. There is well-known link between gout and habitual intake of meat and seafood, however the association between hyperuricemia and micro-and macro-nutrient intake has not been established.

**Methods:**

We studied associations between intakes of food categories, macro-and micronutrients and serum uric acid (SUA) levels in two cross-sectional surveys of Caucasian adults deriving from different food traditions: Australian Diabetes, Obesity and Lifestyle Study 1999/00 (n=9734, age 25–91) and Tromsø Study 4 1994/95 (n = 3031, age 25–69). Dietary intake was calculated from self-administered Food Frequency Questionnaires. In some analyses we stratified according to abdominal obesity status and gender.

**Results:**

In both cohorts, lower levels of SUA were found in subjects with higher consumption of carbohydrates, calcium and vitamin B2, while higher fat intake was associated with higher SUA, after adjustment for age, body mass index, estimated glomerular filtration rate, physical activity, total energy intake, use of diuretics, presence of hypertension, diabetes and gout. Among individual food items, high consumption of dairy products*,* high-fibre bread, cereals and fruits were associated with lower SUA in most subject groups while consumption of meat, eggs, beer and spirits, but not wine, with elevated levels.

**Conclusions:**

Healthy food choices with high intake of carbohydrates, dairy products, fiber and micronutrient-rich foods, and limited intake of fat, beer and spirits, might be recommended to prevent high SUA. Dietary factors seem to have qualitatively similar impact on SUA in obese and non-obese men and women from Australia and Norway.

**Electronic supplementary material:**

The online version of this article (doi:10.1186/s12937-015-0032-1) contains supplementary material, which is available to authorized users.

## Background

Hyperuricemia is a modifiable condition that can lead to gout, and may be an independent risk factor for cardiovascular events [1-4], hypertension [5,6], diabetes [7] and renal disease [8,9]. Reduction of serum uric acid (SUA) level might therefore be an important factor in the prevention of these conditions, with lifestyle modification preferable to pharmacological intervention.

There is long-established link between gout and habitual intake of purine rich foods, in particular meat and seafood [10], however it is only relatively recently that data from the Third National Health and Nutrition Examination Survey provided epidemiological evidence that a purine-rich diet was associated with higher SUA levels in a representative sample of the US population enriched for African-American and Mexican ethnicities. High levels of meat and seafood consumption and moderate to high intake of beer and liquor were associated with higher SUA levels in this study, whereas an inverse association with dairy consumption was observed [11,12].

There is substantial variation in the distribution of measured SUA levels and in the predisposition to hyperuricemia-associated conditions across different races/ethnicities and genders, which could partially be explained by genetic differences [13,14]. Dietary patterns also vary across countries and ethnicities [15] and predominance of unhealthy food choices seem to be in accordance with disproportional burden of “life style diseases” in some populations [16,17].

The present paper explores the relationship between SUA and dietary factors in more detail by examining the association between SUA and intakes of macro- and micronutrients estimated from dietary surveys, in addition to consumption of different food categories, and by comparing data from two large cohorts of homogenous ethnicity but from different geographical locations and food traditions (Australia and Norway). As obesity and male gender are factors typically associated with both elevated SUA and preference for energy-dense foods [18-22] , the cohorts were also analyzed stratified according to abdominal obesity status and gender.

We hypothesized that individual food categories might be differently associated with SUA in Australia and Norway, reflecting differences in food processing and preparation, and the frequency with which certain foods are consumed (especially meat and dairy products). By assessing actual macro-and micro-nutrient intakes it should be possible to make a more objective assessment of dietary factors associated with elevated SUA, allowing our results to be generalized more broadly to other (Caucasian) populations.

## Methods

### Study populations

#### Australian cohort

Australian Diabetes, Obesity and Lifestyle Study (AusDiab) is a population-based survey of adults aged ≥25 years. The present analysis used cross-sectional baseline data (1999/00). Details of survey methods and sample selection have been previously described [23]. In brief, a representative sample of Caucasian Australian population was obtained using a stratified cluster sampling method (n = 11,247). Participants missing uric acid measurement (n = 2) and those with known or suspected myocardial infarction (n = 548) or ischemic stroke (n = 380) prior to the investigation were excluded from the analysis, the latter because of the expected changes in their diets. Data from the food frequency questionnaire (FFQ) were missing for 751 participants, resulting in 9734 valid subjects (5439 females and 4295 males).

#### Norwegian cohort

The Tromsø Study is a population-based, prospective study of residents of the municipality of Tromsø, Norway. In the 4th survey (1994–95), 37558 residents were invited and 27158 (72%) participated in screening. Data on nutrient intake were available for 17265 participants (men and women aged 25–69 years old only). All persons 55–74 years old, and a 5-10% sample of other age groups (n = 9057) were asked to return for a more comprehensive examination 4 to 12 weeks later of which 7965 participated. Measurements of serum uric acid were undertaken on this cohort (n = 7490). Among those who attended the second visit, 6902 (87 %) participated in the full second-visit examination which included anthropometric examination, blood pressure measurements, advanced urinalysis and full medication records. Of these, 41 were excluded due to lack of written consent. Participants missing uric acid measurement (n = 404) and nutrient data (n = 3470), two outliers with serum uric acid concentration deviating by five times the standard deviation, as well as those with known myocardial infarction (n = 405) and ischemic stroke (n = 101) prior to the investigation were excluded from the analysis, the latter because of the expected changes in their diets. This resulted in 3031 valid subjects (1471 females and 1560 males).

### Questionnaires

The questions used for dietary assessment in the Tromsø Study can be found at http://tromsoundersokelsen.uit.no/tromso/. The AusDiab study collected dietary information using FFQ developed by the Cancer Council of Victoria [24].

The two data sets were handled separately because of differences in survey dates, distribution of food intakes and SUA standardization. Nutrient intake was calculated according to conversion methods standardized to each population [25-28].

### Laboratory analyses and physical examinations

Physical examination of participants of AusDiab included blood pressure measurement, assessment of waist circumference, height and weight, collection of blood following overnight fast and a random spot urine specimen. All non-pregnant participants and those not receiving treatment for diabetes underwent a standard 75 g oral glucose tolerance test. Blood and urine samples were transferred to a central laboratory for analysis (HITECH pathology, Clayton Victoria). SUA was measured by enzymatic methods (Olympus AU600 analyser; Olympus Optical Co. Ltd, Tokyo, Japan). Details of other laboratory methods are published elsewhere [29]. Trained interviewers administered standardized questionnaires that collected information regarding demographic characteristics, smoking habits, physical activity, existing health conditions and current use of medications. Information regarding frequency and volume of alcohol consumption was recorded by the FFQ [30]. The intake of alcohol was categorized as “>10 g/day”, “5-10 g/day”, “less than 5 g/day” and “Never”. The first three categories correspond to “>1 standard drink per day”, “ ½-1 standard drink per day” and “<½ standard drink per day” respectively according to Standard 2.7.1 of Food Standards Australia New Zealand.

Physical examination of participants of The Tromsø study included blood pressure measurement, assessment of waist circumference, height and weight by trained nurses and collection of random blood and morning midstream urine specimen. Details of survey methods, sample selection and analyses have been previously described [26,31]. In the Tromsø Study, SUA was measured with COBAS® instruments (Roche diagnostics, Switzerland) using the uricase/ PAP method.

### Definitions

Abdominal obesity was defined as waist circumference >102 cm (40 inches) for men and >88 cm (35 inches) for women, or Body Mass Index (BMI) of more than 30 kg/m^2^. In the Tromsø Study 4, diabetes was defined as self-reported diabetes, or self-reported use of insulin or anti-diabetic medications, or random blood glucose >10.0 mmol/l or HbA1c ≥ 6.5% in blood samples collected at visit 2 investigation. In AusDiab, diabetes was defined as self-reported diabetes; or self-reported use of insulin or oral glucose agents; or fasting plasma glucose ≥ 7.0 mmol/l; or post-load plasma glucose ≥ 11.1 mmol/l; or HbA1c ≥ 6.5 %. Hypertension was defined in both studies as mean systolic blood pressure ≥140 mmHg, or mean diastolic blood pressure ≥90 mmHg, or self-reported use of antihypertensive medications. Use of diuretics in the Tromsø Study was defined as self-reported use of bendroflumethiazide, polythiazide, trichlormethiazide, chlortalidone, mefruside, furosemide, spironolactone, hydrochlorothiazide in combination with potassium-sparing agents, or non-specified diuretic during the last week prior to examination. Use of anti-gout medications in the Tromsø Study was defined as self-reported use of allopurinol, tisopurine, febuxostat, probenecid, sulfinpyrazone, benzbromarone, isobromindione, colchicine, cinchophen, urate oxidase or pegloticase. Data on diuretics and anti-gout medications were not collected as part of the AusDiab study; instead, participants were asked “Have you ever suffered from gout”. Participants were defined as physically active if they reported leisure time activity of at least one hour of sweat- or dyspnoea-inducing exercise per week [32]. Smoking habits were divided into current smokers and currently non-smokers.

### Statistical Analyses

UNIANOVA procedure in SPSS which provides regression analysis and analysis of variance for one dependent variable by one or more factors and/or variables, was used to compare mean SUA values across categories of food intake and across quartiles of nutrient intake. Polynomial contrasts were used to test for linear trends across the adjusted mean values. Least significant difference t test within UNIANOVA procedure was used to compare the difference in SUA level between highest versus lowest category of food intake or highest versus lowest quartile of nutrient intake. For the AusDiab, the models were adjusted for age, sex, BMI, eGFR (CKD-EPI), presence of hypertension, presence of diabetes, alcohol intake above 10 g/day, self-reported history of gout at baseline, 1 h or more of vigorous physical activity in the past week and daily energy intake. For the Tromsø Study, the models were adjusted for age decade, sex, BMI, eGFR (CKD-EPI), presence of hypertension, presence of diabetes, alcohol intake above 10 g/day, self-reported use of anti-gout medications, self-reported use of diuretics, 1 h or more of vigorous physical activity in the past week and daily energy intake. All p-values were two-sided, p < 0.05 considered statistically significant. The statistical analyses were performed using IBM SPSS version 19.0 (www.ibmspss.com/).

### Ethics

Both studies were conducted according to the guidelines laid down in the Declaration of Helsinki. The Tromsø Study was performed in collaboration with The National Health Screening Service and was approved by The Regional Committee for Medical Research Ethics, Tromsø, Norway. The AusDiab Study was approved by the International Diabetes Institute ethics committee (Melbourne, Australia). All participants gave their written informed consent.

## Results

### Population characteristics

Selected baseline characteristics of the study participants are presented in Table 1. Norwegian participants were older with higher prevalence of hypertension and smoking, higher average levels of HbA1c, SUA, total cholesterol, HDL cholesterol and higher percentage of energy intake (E%) from carbohydrates. On the other hand, body mass index, waist circumference, total energy intake in women, E% from fat and protein intakes and reported alcohol intake above 10 g per day were lower compared to their Australian counterparts.

**Table 1:** Baseline characteristics of the study participants

	AusDiab 1999/00				The Tromsø study 1994/95			
	Males (N = 4295)		Females (N = 5439)		Males (N = 1560)		Females (N = 1471)	
	Mean/Median/%	SD/IQR	Mean/Median/%	SD/IQR	Mean/Median/%	SD/IQR	Mean/Median/%	SD/IQR
Age, years	49	40-60	49	40-60	57	51-62	58	54-63
Never smoker, %	47.3		61.9		22.1		39.6	
Former smoker, %	34.5		23.6		42.9		26.6	
Current smoker, %	18.2		14.6		35.0		33.8	
Physically active, %	29		20		33		18	
Waist circumference, cm	97	11	85	13	94	9	83	11
Body Mass Index, kg/m^2^	27	4	27	6	26	3	25	4
Hypertension, %	33.5		26.6		53.6		45.3	
Use of anti-hypertensives, %	12.0		14.2		8.6		8.1	
HbA1c, %	5.2	0.6	5.2	0.6	5.4	0.5	5.4	0.5
Total Cholesterol, mmol/l	5.7	1.0	5.7	1.1	6.4	1.2	6.6	1.4
HDL cholesterol, mmol/l	1.27	0.32	1.56	0.38	1.36	0.38	1.66	0.43
Triglycerides, mmol/l	1.40	0.98-2.10	1.16	0.80-1.70	1.41	0.98-2.04	1.13	0.82-1.61
eGFR (CKD-EPI formula)	80	12	74	12	97	13	96	14
Albumin/creatinine ratio	0.47	0.35-0.76	0.61	0.46-1.03	0.49	0.33-0.84	0.57	0.39-0.90
Serum Uric Acid , μmol/l	344	76	25	72	360	85	269	66
Alcohol: Never	9.2		17.6		30.7		49.8	
Alcohol: <5 g/day	23.0		41.5		43.0		41.4	
Alcohol: 5–10 g/day	12.3		12.8		19.3		7.3	
Alcohol: >10 g/day	55.5		28.1		7.0		1.6	
Total energy intake, kJ/day	9490	3599	7178	2958	9039	1902	6687	1637
Protein, Energy %	19	3	20	3	17	2	17	2
Fat, Energy %	37	5	35	6	28	6	30	6
Carbohydrate, Energy %	45	6	46	6	54	6	52	6

The categorical data are presented as percentage. The continuous data are presented as mean when normally distributed or median when not. SD, standard deviation; IQR, interquartile range.

AusDiab 1999/00 and The Tromsø Study 1994/95.

### Food categories

**Figure 1:**
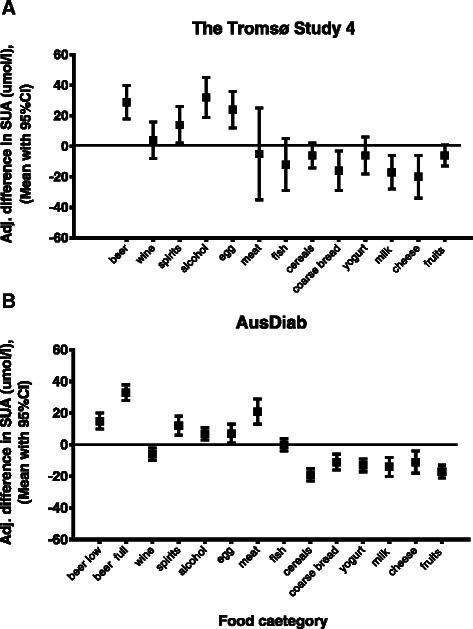
Mean difference in serum uric acid between highest and lowest categories of food intake in the participants of AusDiab and The Tromsø Study.Estimated marginal means and 95 % CI for the difference in SUA between the highest and lowest categories of food intakes. The Tromsø Study **(A)** models were adjusted for age decade, BMI, eGFR, presence of hypertension, presence of diabetes, use of diuretics, use of anti-gout medication, sweat or dyspnoea-inducing physical activity of ≥1 h/week, alcohol intake >10 g/day (except for alcohol variables) and daily energy intake. The AusDiab **(B)** models were adjusted for age, BMI, eGFR, presence of hypertension, presence of diabetes, alcohol intake >10 g/day (except for alcohol variables), self-reported history of gout at baseline, vigorous physical activity of ≥1 h in the past week and energy intake. Least significant difference t-test was used to calculate p-values in pairwise comparisons. The intake categories compared for the Tromsø Study 4: beer: <1 vs. >5 glasses/fortnight; wine: <1 vs. >6 glasses/fortnight; spirits: <1 vs. >6 glasses/fortnight; alcohol 0 vs. >10 g/day; eggs never vs. >1/week; meat: <1 vs. >2 times/week; fish: <1 vs. >3 times/week; cereals: never vs. >1 times/week; coarse bread: <3 vs. >6 slices/day; yogurt: never vs. >3 times/week; milk: never vs. >2 glasses/day; bread with cheese: never vs. >2 slices/day; fruits: <1 vs. >4 pieces/day; for the AusDiab: beer (low and full strength): 0 vs. >2 days/week; wine: 0 vs. >2 days/week; spirits: 0 vs. >2 days/week; alcohol 0 vs. >10 g/day; eggs never vs. >2/week; meat: <1 vs. >6 times/week; fish: <1 vs. >3 times/week; cereals: never vs. >1 times/week; coarse bread: <2 vs. >4 slices/day; yogurt: never vs. >4 times/week; milk: never vs. >2 glasses/day; cheese: never vs. >4 times/week; fruits: <1 vs. >2 pieces/day.

Figure 1 shows summary results for the relationship between food and alcohol intake and SUA levels in AusDiab and the Tromsø Study cohorts. After adjustment for confounders, SUA levels were significantly higher amongst the highest versus lowest consumers of alcohol in both cohorts (>10 g alcohol per day versus non-drinkers). When different alcohol types were analyzed separately, the highest consumers of beer and spirits had significantly higher SUA levels in both cohorts, however the highest consumers of wine in the Australian cohort had significantly lower SUA. SUA levels were significantly higher amongst the highest versus lowest consumers of eggs in both cohorts. Meat, cereal and yoghurt intake were significantly associated with SUA level in the Australian cohort only. The highest intake of coarse bread, milk and cheese were associated with significantly lower SUA levels in both cohorts. Detailed results of analyses stratified for abdominal obesity and gender are provided as (Additional file 1: Tables S1), (Additional file 2: Tables S2), (Additional file 3: Tables S3), (Additional file 4: Tables S4). Key results from the supplementary tables are summarized below.

#### *Meat*

There was strong direct relationship between intake of meat and SUA in the non-obese participants of the Australian cohort. After adjustment for age or other confounders, a significant relationship was also observed for obese women. The Norwegian cohort had significantly lower meat consumption (99 % of the study participants eat meat for dinner three or fewer times per week) and no association between SUA and consumption of meat was observed.

#### *Fish*

Consumption of fish was not associated with higher levels of SUA in the participants of our studies. Moreover, high intake of fish was associated with significantly lower SUA levels among the obese females from AusDiab (Additional file 1: Table S1). It is, however, not clear, what fish species/preparations dominated in the diet in the Australian study.

#### *Eggs*

The highest category of egg consumption (more than one per week) was associated with highest average level of SUA in all the groups of the Tromsø cohort. This was significant in men, and after multiple adjustment, in non-obese men and obese women. The results in the Australian cohort were similar.

#### *Dairy products*

Drinking milk was associated with considerably lower SUA level in obese women and non-obese men from the Tromsø cohort and in all the groups from AusDiab. Yoghurt consumption in the Australian cohort was greater and had strong inverse relationship with SUA in all subgroups after multivariate adjustment (p < 0.001), with the exception of obese men.

Intake of cheese had a significant inverse relationship with SUA in non-obese men, and similar but non-significant trends in other groups, in the Australian cohort. In the Norwegian study, the number of slices of bread with cheese consumed per day had also a clear inverse relationship with SUA, which was significant in all the groups except non-obese women.

#### Cereals and bread

Cereals had a significant inverse relationship with SUA after multivariate adjustment in all the groups in the Australian cohort (p < 0.001). In the Norwegian cohort, only obese men – and to a lesser extent obese women – showed trends in the same direction (p = 0.126 and p = 0.172, respectively). Consumption of high-fiber bread was associated with significantly lower levels of SUA in the non-obese men from the Tromsø study (p = 0.015). High consumption of high-fiber bread in AusDiab was associated with significantly reduced SUA levels in non-obese men and obese women, with similar trends in the other groups.

#### *Fruits and vegetables*

Higher consumption of fresh fruit appeared to be associated with lower SUA in all groups of the Australian cohort and in the obese females in the Norwegian cohort. No significant relationship was observed between serum uric acid and consumption of vegetables in either of the datasets (data not shown).

#### *Alcohol*

Alcohol consumption had direct relationship with SUA in both Australian (Additional file 3: Tables S3) and Norwegian (Additional file 4: Tables S4) men. The threshold at which a significant increase in SUA was observed was 5 g/day with full-strength beer having the most prominent effect among alcoholic beverages. Similar trends were observed for low-strength beer, such that higher levels of consumption were associated with higher mean SUA in all groups except for obese females, uncontrolled for intake of other alcoholic beverages (data not shown). Consumption of wine was not associated with higher SUA in any of the subgroups in either cohort. There were no significant associations between intake of alcohol and SUA for women in the Tromsø Study except for positive association between consumption of beer in the subgroup of non-obese women.

### Macronutrients

**Figure 2:**
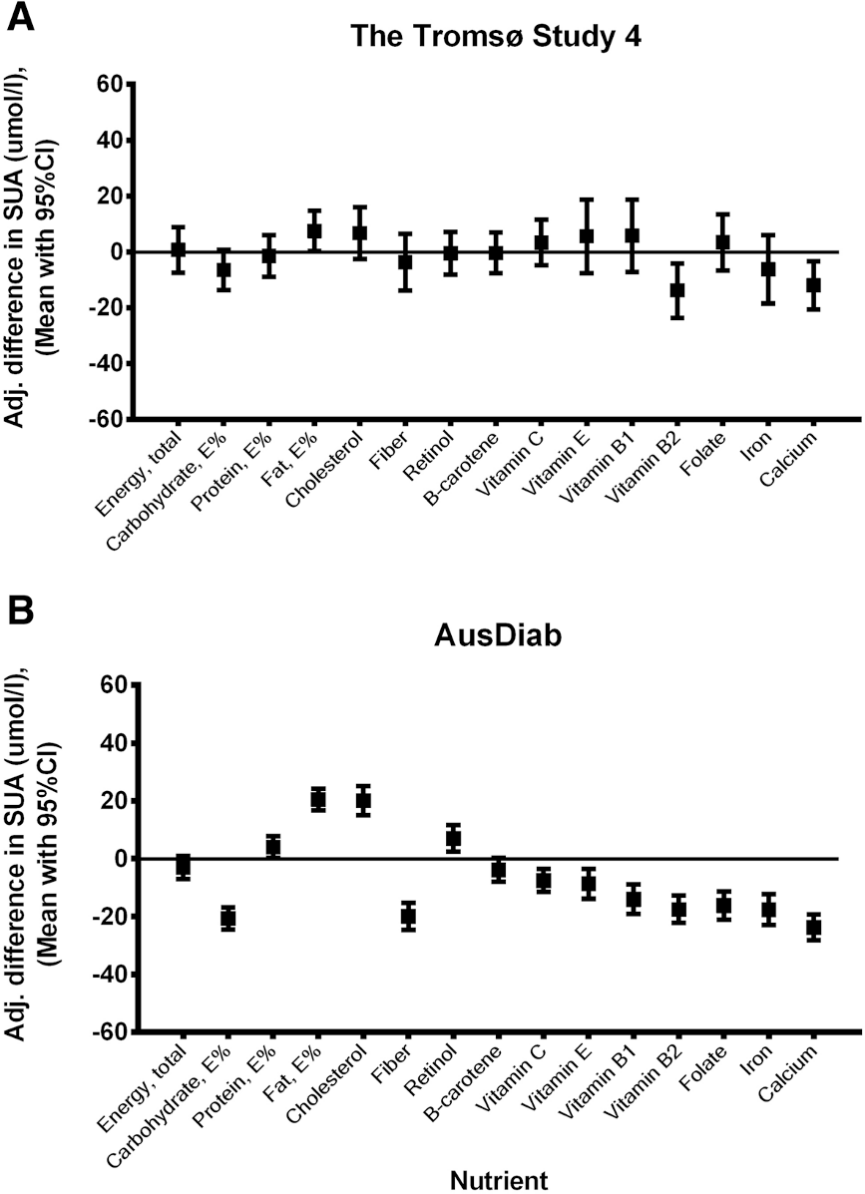
Mean difference in serum uric acid between highest and lowest quartiles of nutrient intake in the participants of AusDiab and The Tromsø Study.Estimated marginal means and 95 % Confidence intervals for the difference in serum uric acid between the highest (Q4) and lowest (Q1) quartiles of nutrient intakes. The Tromsø Study **(A)** models were adjusted for age decade, BMI (continuous), eGFR (CKD-EPI, continuous), presence of hypertension, presence of diabetes, alcohol intake above 10 g/day, use of diuretics, use of anti-gout medication, sweat or dyspnoea-inducing physical activity of 1 h and more per week and daily energy intake (kj/day, continuous). The AusDiab **(B)** models were adjusted for age (continuous), BMI (continuous), eGFR (CKD-EPI, continuous), presence of hypertension, presence of diabetes, alcohol intake above 10 g/day, self-reported history of gout at baseline, 1 h or more of vigorous physical activity in the past week and daily energy intake (kj/day, continuous). Least significant difference ttest was used to calculate p-values in pairwise comparisons.

Figure 2 shows the adjusted mean difference in SUA level between the highest and lowest quartiles of macro- and micronutrient intake. Additional file 5: Tables S5 and Additional file 6: Tables S6 provide detailed results across obesity - and gender-specific quartiles of nutrient intake, for each subgroup of obese and non-obese men and women.

Total energy consumed per day (Figure 2) was not significantly associated with serum uric acid levels in either cohort (Linear trend:–2.15, p = 0.143 in AusDiab and 0.41, p = 0.893 in the Tromsø Study). In contrast, higher consumption of carbohydrates (both in g/day and as proportion of energy, with or without multivariate adjustment) was strongly associated with lower levels of SUA in AusDiab (Linear trend: −15.17, p < 0.001). Similar results but with marginal significance were observed in the Tromsø Study (Linear trend: −4.94, p = 0.057) . In both datasets, the highest quartiles of absolute carbohydrate intake were associated with up to a 35 μmol/l reduction in SUA level compared to the lowest quartiles (Additional file 5: Tables S5 and Additional file 6: Tables S6). Intake of sugar was also associated with significant reduction in SUA in the Australian study (Additional file 1: Tables S1). There was a strong, significant inverse relationship between consumption of fiber and SUA in all AusDiab gender-and obesity-subgroups (15-27 μmol/l reduction in quartile 4 vs.1). No such association was seen in participants from Norway where the maximal daily fiber intake was at least two fold lower than the corresponding value in AusDiab (Figure 2, Additional file 5: Tables S5 and Additional file 6: Tables S6).

Protein intake was not significantly associated with SUA level in either cohort (Linear trend: −0.12, p = 0.966 in The Tromsø study and 2.29, p = 0.095 in AusDiab). Dietary fat, however, was significantly associated with higher SUA in both datasets, particularly when expressed as percentage of the total energy consumed (Linear trend: 4.29, p = 0.102 in The Tromsø study and 14.94, p < 0.001 in AusDiab).

### Micronutrients

Higher calcium intake was strongly associated with lower SUA levels both in the Tromsø study (Linear trend:–9.05, p = 0.004) and in AusDiab (Linear trend:–17.54, p < 0.001). The minimal intake associated with statistically significant serum uric acid reduction was at a level of approximately 1000 mg of calcium per day for men and 650 mg per day for women in both datasets (Additional file 5: Tables S5 and Additional file 6: Tables S6). Among vitamins, riboflavin (vitamin B2) derived from food had the strongest inverse association with SUA level, significant in both cohorts (The Tromsø study: –9.71, p = 0.006 and AusDiab: –12.42, p < 0.001). The average reduction of SUA was significant in most groups when intakes were above 2 mg per day (the recommended daily dose is 1.3 mg for men and 1.1 mg for women [33]). Consumption of iron above 11 g per day was associated with considerable reduction of uric acid level (by 10-30 μmol/l) in all the groups in AusDiab (Figure 2 and Additional file 5: Tables S5. Linear trend:–12.93, p < 0.001 for pooled sample). A trend in the same direction was noted in the Tromsø study but statistical significance was not reached (Linear trend:–5.10, p = 0.260). Higher dietary intakes of thiamin (vitamin B1) and folate (vitamin B9) were associated with considerable reduction in SUA level but only in the Australian cohort (Linear trends:–9.97, p < 0.001 and–12.82 and p < 0.001 respectively). The amounts consumed by the majority of Norwegian participants were below values for the lowest consumption quartile showing a difference in SUA in AusDiab.

## Discussion

The present study investigating two large, Caucasian population samples from Australia and Norway shows that SUA has similar relationship with dietary factors across genders and appears not to be qualitatively affected by the presence of abdominal obesity. In both cohorts, higher intakes of carbohydrate-, calcium- and vitamin B2-rich foods were associated with lower levels of SUA while dietary fat showed an opposite association. High consumption of fat, but not protein or total energy intake, was associated with higher SUA in this study. This is consistent with previous studies linking a low-carbohydrate, high fat calorie-restricted diet with significant elevation of SUA [34], and low-fat high-protein and low-fat high-carbohydrate calorie-restricted diets with a decrease in SUA in small groups of healthy volunteers [35]. Therefore, the adverse association of meat consumption and SUA level may be at least partly attributable to the quality and preparation methods of meat and its saturated fat content, rather than the quantity of meat consumption *alone*.

In a German study, high meat consumption was linked to adherence to generally unhealthy food patterns (the processed foods pattern), along with high intake of eggs, refined grains, beer and sweets [36]. This study also found significantly higher SUA concentrations in the highest quintile of the processed food consumers. Our finding of adverse associations between SUA and meat, eggs and beer, in contrast to beneficial associations with higher intakes of cereals, fruits and dairy products are in keeping with this report. Our results are also consistent with a recent study linking adherence to a Mediterranean diet, characterized by high intakes of cereals, fruits and vegetables, with lower SUA [37]. Lacto-vegetarian diets are associated with many health benefits largely attributable to a higher content of fiber and some vitamins [38]. In line with the results of our study, lacto-vegetarian diet was associated with significantly lower SUA compared to omnivorous eating in healthy Chinese [39]. Moreover, vegans were found to have highest serum concentrations of uric acid compared to vegetarians, meat-eaters and fish-eaters in a selection of participants from Oxford cohort of the European Prospective Investigation into Cancer and Nutrition [40].

The inverse associations of dietary calcium and vitamin B2 with SUA level in the present study supports a previous report describing lower SUA with increasing consumption of dairy products [11]. This might indicate a direct (e.g. uricosuric) effect of dairy proteins [41]. An alternative explanation is that lower milk consumption presents an indirect evidence of compromised gastrointestinal health, such as lactose or milk protein intolerance, leading to higher SUA through impaired intestinal uricolysis [42]. Whereas a large number of epidemiological studies have reported on possible beneficial effects of dairy consumption on diabetes risk factors, pointing to increased satiety or a protective role of calcium and magnesium [43-45], recent studies have implicated changes in gut microbiota in the development of obesity, glucose intolerance and hypertension [46,47], factors clustering with hyperuricemia [48]. This is consistent with the findings of Choi et al. that, of all dairy products, yoghurt showed the greatest benefits with respect to SUA levels [11]. In keeping with this hypothesis, the stronger inverse association between yoghurt consumption and SUA in the Australian cohort could be related to a more widespread use of live probiotic bacteria in yoghurt preparations in contrast to Norway at the time of study baseline. Finally, the inverse association between calcium intake and SUA could also be related to increased calcium excretion and thus reabsorption of phosphate and upregulation of renal sodium-phosphate cotransporters, which are PTH- and 1.25 (OH)2 vitamin D-dependent. These transporters co-localize with uric acid transporters (URAT1) in the proximal renal tubule. Animal experiments have demonstrated that interactions between tubular anion exchangers and urate transporters are possible, as common binding sites and transport proteins are involved [49,50].

Dietary fiber, iron, vitamins B1 (thiamin) and B9 (folate) also showed a strong beneficial association with hyperuricemia but only in the Australian cohort. One explanation for the difference between the countries is generally lower consumption of these micronutrients among Norwegian study participants. Alternatively, the strong protective association seen in Australian groups could be a reflection of higher consumption of certain beneficial food types, such as bread and cereals fortified with vitamins B1 and B9, and lean red meat, whereas in Norway fortification is not a common practice and meat is consumed less frequently. It is also possible that the protective effect of fiber is only present when associated with higher intake of fats, due to proposed alterations of intestinal absorption of lipids mediated by dietary fiber, [51] and therefore fiber was not significantly associated with SUA in the Tromsø cohort with relatively low consumption of fat. High intake of the vitamins might also be of protective importance because of their role in various processes leading to salvage of purines, such as synthesis of nucleic acids. Alternatively, urine alkalization achieved through consumption of vegetable-fruit food materials rich in these micronutrients might be responsible for lowering of SUA through its augmented excretion [52].

In their analysis of the United States population, Choi et al. found that higher serum levels of vitamin A were associated with increasing SUA in both males and females, and suggest that supraphysiological vitamin A supplementation may be contributing to high rates of gout and hyperuricemia in the United States [53]. In contrast, the authors observed an inverse association between SUA and β-carotene, which is a precursor to Vitamin A but largely free of its toxicity. We also observed some evidence of a detrimental effect of vitamin A intake in the AusDiab cohort. β-carotene supplementation is reported to lower SUA levels [54], but we did not observe lower SUA at higher quartiles of β-carotene intake. In terms of Vitamin C, both randomised trials and observational studies have shown that high intake of vitamin C has a beneficial effect on SUA levels and risk of gout, possibly due to uricosuric effects mediated by inhibition of URAT1 and/or sodium-dependent anion cotransport [55,56]. In our analysis, higher intake of vitamin C was associated with lower SUA levels only in females in the AusDiab.

Our study had several limitations. Firstly, the information on dietary habits was collected by self-administered FFQ which are believed to be more prone to errors and bias compared to more comprehensive dietary survey methods [57]. Furthermore, in the case of the Tromsø study, the questionnaires had no information on the sizes of the portions, which were instead stipulated for each gender based on data from previous dietary history surveys in northern Norway. The reproducibility of similar FFQ used in the Tromsø Study 3 was assessed by comparing answers from 201 men and women to identical questions posed in two surveys one year apart and the concordance between the results was high, both concerning food categories most commonly eaten and the frequency with which the food categories were consumed [58]. A limited validation study comparing short FFQ with a dietary history interview 2 years later in 528 participants of The Tromsø Study 2 reported that short FFQ performed well with respect to mean intake of food items used daily in easily recorded units (slices of bread, glasses of milk) but that concordance for other food categories was less satisfactory [59]. In the case of the AusDiab study, the FFQ is based on the Melbourne Collaborative Cohort Study conducted in the late 1980s, and is based on weighted food records from adults 40–69 years born in Australia, Italy or Greece. It therefore may not contain all relevant food items for AusDiab participants, however subsequent validation of the FFQ found that it performed as well as other validated FFQs [60,61]. To reduce this uncertainty regarding energy consumption we performed analyses after stratification according to the obesity status of the participants. The data on alcohol consumption might also be inaccurate to some degree, as under-reporting of alcohol intake is a common phenomenon, particularly among women and individuals with a history of alcohol abuse [62]. The potential for inaccuracy in the measurement of SUA is also a limitation of our study. SUA was measured in random blood samples in The Tromsø Study and in fasting blood samples in AusDiab. Furthermore, alcohol use, some medications and vitamin C concentration can affect SUA measurement [63,64]. The strength of this study was that we sampled data from two large, Caucasian populations, with different cultural and geographic background and for the first time analyzed intakes of micro- and macro-nutrients in addition to food categories. Furthermore, we had the possibility to adjust the results for variations in age, BMI, physical activity, eGFR, presence of diabetes, hypertension, use of diruretics and gout.

## Conclusions

Healthy food choices with high intake of carbohydrates, dairy products, fiber and micronutrient-rich foods and limited intake of fatty meats, eggs, beer and spirit might be recommended to lower SUA levels. The possible beneficial effects of nutritional supplements, particularly calcium, fiber, iron and vitamins B2, B1, B9, C and E on the level of urecimia need to be evaluated in interventional studies. The relationship between the dietary factors and SUA appears similar in both genders and not qualitatively affected by the presence of abdominal obesity.

## Additional files

Additional file 1: Table S1. Mean serum uric acid (SUA) in males and females, according to intake of individual foods and by presence of central obesity: AusDiab 99/00. Additional file 2: Table S2. Mean serum uric acid (SUA) in males and females, according to intake of individual foods and by presence of central obesity: The Tromsø Study 94/95. Additional file 3: Table S3. Mean serum uric acid (SUA) in males and females, according to intake of alcohol and by presence of central obesity: AusDiab 99/00. Additional file 4: Table S4. Mean serum uric acid (SUA) in males and females according to intake of alcohol and by presence of central obesity: The Tromsø Study 94/95. Additional file 5: Table S5. “Serum Uric Acid in Gender- and Obesity Group-specific quartiles of Nutrient Intake. The AusDiab 1999/00”. Additional file 6: Table S6. “Serum Uric Acid in Gender- and Obesity Status-specific quartiles of Nutrient Intake. The Tromsø Study 1994/95’.

## Abbreviations

SUA: Serum uric acid
FFQ: Food Frequency Questionnaire
AusDiab: Australian Diabetes, Obesity and Lifestyle Study
BMI: Body Mass Index
eGFR: Estimated glomerular filtration rate
