# Supplementary material for: Cross-sectional analysis of nutrition and serum uric acid in two Caucasian cohorts: the AusDiab Study and the Tromsø study
Source: Nutr J. 2015 May 14;14:49. doi: 10.1186/s12937-015-0032-1 (PMC4459487; doi:10.1186/s12937-015-0032-1)
Supplement: Additional file 1: Table S1. — Mean serum uric acid (SUA) in males and females, according to intake of individual foods and by presence of central obesity: AusDiab 99/00. [file 12937_2015_32_MOESM1_ESM.docx]

**Supplementary Table 1. Mean serum uric acid (SUA) in males and females, according to intake of individual foods and by presence of central obesity: AusDiab 99/00**

|  | **Males** | | | | | | | | **Females** | | | | | | | |
| --- | --- | --- | --- | --- | --- | --- | --- | --- | --- | --- | --- | --- | --- | --- | --- | --- |
|  | **No central obesity** | | | | **Central obesity** | | | | **No central obesity** | | | | **Central obesity** | | | |
|  |  |  | **SUA, µmol/l** | |  |  | **SUA, µmol/l** | |  |  | **SUA, µmol/l** | |  |  | **SUA, µmol/l** | |
|  | **N** | **%** | **mean** | **SD** | **N** | **%** | **mean** | **SD** | **N** | **%** | **mean** | **SD** | **N** | **%** | **mean** | **SD** |
| Fish (any)^1^, times per week | | | | | | | | | | | | | | | | |
| <1 | 737 | 25 | 327 | 71 | 330 | 24 | 381 | 76 | 830 | 24 | 229 | 59 | 486 | 24 | 298* | 75 |
| 1 | 954 | 33 | 331 | 74 | 467 | 33 | 371 | 75 | 1033 | 30 | 232 | 60 | 555 | 28 | 296 | 75 |
| 2-3 | 849 | 29 | 332 | 73 | 425 | 30 | 369 | 76 | 1059 | 31 | 233 | 61 | 591 | 30 | 282 | 74 |
| >=4 | 354 | 12 | 333 | 67 | 179 | 13 | 364 | 80 | 530 | 15 | 234 | 62 | 355 | 18 | 286 | 76 |
| Meat (unprocessed)^2^, times per week | | | | | | | | | | | | | | | | |
| <1 | 82 | 3 | 292*** | 66 | 22 | 2 | 348 | 62 | 152 | 4 | 220*** | 59 | 48 | 2 | 277* | 87 |
| 1-3 | 580 | 20 | 324 | 73 | 263 | 19 | 372 | 82 | 792 | 23 | 230 | 60 | 413 | 21 | 284 | 78 |
| 4-6 | 1339 | 46 | 330 | 71 | 621 | 44 | 371 | 75 | 1636 | 47 | 231 | 61 | 897 | 45 | 291 | 72 |
| >=7 | 893 | 31 | 338 | 73 | 495 | 35 | 373 | 76 | 872 | 25 | 239 | 61 | 629 | 32 | 295 | 77 |
| Eggs per week | | | | | | | | | | | | | | | | |
| 0 | 171 | 6 | 328** | 67 | 73 | 5 | 362 | 77 | 222 | 6 | 235* | 66 | 113 | 6 | 304** | 84 |
| <1 | 783 | 27 | 326 | 70 | 384 | 27 | 367 | 77 | 1175 | 34 | 228 | 58 | 631 | 32 | 284 | 72 |
| 1-2 | 1306 | 45 | 331 | 72 | 580 | 41 | 375 | 76 | 1636 | 47 | 234 | 61 | 970 | 49 | 290 | 77 |
| >=3 | 634 | 22 | 335 | 76 | 364 | 26 | 374 | 76 | 419 | 12 | 231 | 64 | 273 | 14 | 302 | 71 |
| Cheese^3^, times per week | | | | | | | | | | | | | | | | |
| 0 | 136 | 5 | 333* | 74 | 61 | 4 | 377 | 87 | 153 | 4 | 241* | 66 | 93 | 5 | 302 | 74 |
| <1 | 350 | 12 | 338 | 71 | 186 | 13 | 376^a^ | 72 | 374 | 11 | 241 | 64 | 225 | 11 | 298 | 75 |
| 1-4 | 1608 | 56 | 332 | 74 | 807 | 58 | 375 | 77 | 1947 | 56 | 230 | 60 | 1141 | 57 | 290 | 74 |
| >=5 | 800 | 28 | 324 | 70 | 347 | 25 | 362 | 74 | 978 | 28 | 230 | 60 | 528 | 27 | 287 | 77 |
| Yoghurt, times per week | | | | | | | | | | | | | | | | |
| 0 | 1037 | 36 | 335** | 74 | 586 | 42 | 373 | 78 | 643 | 19 | 245*** | 67 | 482 | 24 | 300** | 75 |
| <1 | 895 | 31 | 332 | 72 | 419 | 30 | 375 | 74 | 1052 | 30 | 229 | 57 | 563 | 28 | 292 | 75 |
| 1-4 | 656 | 23 | 327 | 69 | 271 | 19 | 371 | 77 | 1118 | 32 | 229 | 59 | 586 | 29 | 286 | 74 |
| >=5 | 306 | 11 | 317 | 72 | 125 | 9 | 361 | 75 | 639 | 19 | 229 | 62 | 356 | 18 | 282 | 75 |
| Milk (any), glasses per day | | | | | | | | | | | | | | | | |
| 0 | 296 | 10 | 339*** | 78 | 129 | 9 | 379* | 83 | 381 | 11 | 232*** | 66 | 170 | 9 | 303* | 82 |
| <1 | 1132 | 39 | 337 | 73 | 609 | 43 | 376 | 78 | 1383 | 40 | 237 | 62 | 831 | 42 | 292 | 75 |
| 1-2 | 1055 | 36 | 326 | 71 | 489 | 35 | 367 | 74 | 1337 | 39 | 227 | 57 | 798 | 40 | 287 | 75 |
| >2 | 411 | 14 | 316 | 65 | 174 | 12 | 368 | 71 | 351 | 10 | 229 | 59 | 188 | 9 | 287 | 72 |
| Cereals, times per week | | | | | | | | | | | | | | | | |
| 0 | 444 | 15 | 342*** | 74 | 237 | 17 | 394*** | 87 | 445 | 13 | 243*** | 66 | 297 | 15 | 304*** | 75 |
| <1 | 534 | 18 | 335 | 71 | 279 | 20 | 378 | 73 | 633 | 18 | 228 | 58 | 360 | 18 | 293 | 77 |
| 1 | 194 | 7 | 336 | 74 | 101 | 7 | 372 | 70 | 246 | 7 | 233 | 62 | 166 | 8 | 295 | 79 |
| >=2 | 1722 | 60 | 325 | 72 | 784 | 56 | 363 | 73 | 2128 | 62 | 230 | 60 | 1164 | 59 | 286 | 74 |
| Bread (high-fibre only)^4^, slices per day | | | | | | | | | | | | | | | | |
| <2 | 1226 | 42 | 338*** | 76 | 665 | 47 | 378 | 77 | 1294 | 37 | 231 | 60 | 718 | 36 | 296* | 76 |
| 2 | 366 | 13 | 330 | 70 | 172 | 12 | 368 | 77 | 872 | 25 | 234 | 61 | 488 | 25 | 284 | 81 |
| 3-4 | 931 | 32 | 325 | 69 | 417 | 30 | 367 | 77 | 1186 | 34 | 232 | 61 | 700 | 35 | 289 | 71 |
| >=5 | 371 | 13 | 318 | 68 | 147 | 10 | 362 | 70 | 100 | 3 | 221 | 60 | 81 | 4 | 291 | 70 |
| Pieces of fresh fruit per day | | | | | | | | | | | | | | | | |
| <1 | 901 | 31 | 337** | 75 | 462 | 33 | 379 | 73 | 675 | 20 | 236*** | 60 | 420 | 21 | 296*** | 72 |
| 1 | 865 | 30 | 332 | 70 | 405 | 29 | 374 | 77 | 1017 | 29 | 228 | 59 | 530 | 27 | 298 | 76 |
| 2 | 678 | 23 | 326 | 73 | 320 | 23 | 367 | 80 | 1026 | 30 | 233 | 62 | 577 | 29 | 289 | 76 |
| >=3 | 450 | 16 | 319 | 69 | 214 | 15 | 358 | 76 | 734 | 21 | 230 | 61 | 460 | 23 | 279 | 74 |

AusDiab participants were asked to recall their usual eating habits averaged over the past 12 months. Intake categories were constructed based on participant responses, to best reflect the patterns of consumption of individual food types as reported by this cohort.

^*^ P-value for linear trend, model adjusted for age (continuous), BMI (continuous), eGFR (CKD-EPI, continuous), presence of hypertension, presence of diabetes, alcohol intake above 10g/day, self-reported history of gout at baseline, 1h or more of vigorous physical activity in the past week, energy intake (kj/day, continuous) *P<0·05, **P<0·01, ***P<0∙001

^1^ Average number of times over the last 12 months that fish (steamed, grilled, baked, fried or tinned) was consumed

^2^ Average number of times over the last 12 months that beef, veal, chicken, lamb and/or pork were consumed

^3^ Average number of times that cheese was consumed over the past 12 months.

^4^ The usual number of slices of bread consumed per day for those who normally eat high fibre white bread, wholemeal bread, rye bread or multi-grain bread, including fresh and toasted bread.
