# Supplementary material for: Cross-sectional analysis of nutrition and serum uric acid in two Caucasian cohorts: the AusDiab Study and the Tromsø study
Source: Nutr J. 2015 May 14;14:49. doi: 10.1186/s12937-015-0032-1 (PMC4459487; doi:10.1186/s12937-015-0032-1)
Supplement: Additional file 2: Table S2. — Mean serum uric acid (SUA) in males and females, according to intake of individual foods and by presence of central obesity: The Tromsø Study 94/95. [file 12937_2015_32_MOESM2_ESM.docx]

**Supplementary Table 2. Mean serum uric acid (SUA) in males and females, according to intake of individual foods and by presence of central obesity: The Tromsø Study 94/95**

|  | **Male** | | | | | | | | **Female** | | | | | | | |
| --- | --- | --- | --- | --- | --- | --- | --- | --- | --- | --- | --- | --- | --- | --- | --- | --- |
|  | **No abdominal obesity** | | | | **Abdominal Obesity** | | | | **No abdominal obesity** | | | | **Abdominal obesity** | | | |
|  |  |  | **SUA, µmol/l** | |  |  | **SUA, µmol/l** | |  |  | **SUA, µmol/l** | |  |  | **SUA, µmol/l** | |
|  | **N** | **%** | **mean** | **SD** | **N** | **%** | **mean** | **SD** | **N** | **%** | **mean** | **SD** | **N** | **%** | **mean** | **SD** |
| Fish, times per week | | | | | | | | | | | | | | | | |
| <1 | 65 | 5 | 360 | 88 | 12 | 4 | 393 | 77 | 57 | 6 | 252 | 46 | 27 | 7 | 315 | 79 |
| 1 | 479 | 38 | 353 | 81 | 94 | 34 | 407 | 110 | 422 | 41 | 255 | 61 | 150 | 38 | 301 | 63 |
| 2-3 | 653 | 52 | 348 | 77 | 157 | 58 | 399 | 91 | 501 | 49 | 257 | 58 | 199 | 51 | 307 | 76 |
| >3 | 52 | 4 | 348 | 81 | 10 | 4 | 405 | 125 | 47 | 5 | 250 | 65 | 18 | 5 | 293 | 74 |
| Meat, times per week | | | | | | | | | | | | | | | | |
| <1 | 83 | 7 | 352 | 89 | 14 | 5 | 397 | 99 | 93 | 9 | 253 | 56 | 31 | 8 | 312 | 79 |
| 1 | 637 | 51 | 348 | 77 | 138 | 50 | 397 | 84 | 585 | 57 | 255 | 62 | 220 | 56 | 297 | 75 |
| 2-3 | 508 | 41 | 351 | 79 | 121 | 44 | 410 | 112 | 336 | 33 | 257 | 54 | 140 | 35 | 309 | 60 |
| >3 | 12 | 1 | 347 | 84 | 2 | 1 | 449 | 35 | 5 | 0.5 | 204 | 45 | 4 | 1 | 334 | 106 |
| Eggs, times per week | | | | | | | | | | | | | | | | |
| never | 71 | 6 | 330* | 75 | 19 | 7 | 369 | 44 | 98 | 9 | 256 | 55 | 26 | 6 | 287* | 67 |
| <1 | 569 | 45 | 348 | 73 | 115 | 42 | 402 | 109 | 483 | 46 | 253 | 60 | 181 | 45 | 300 | 67 |
| 1 | 439 | 34 | 351 | 81 | 84 | 31 | 395 | 90 | 364 | 35 | 258 | 60 | 148 | 37 | 309 | 76 |
| >1 | 196 | 15 | 362 | 88 | 57 | 21 | 423 | 98 | 101 | 10 | 261 | 55 | 49 | 12 | 312 | 69 |
| Bread with cheese, slices per day | | | | | | | | | | | | | | | | |
| never | 93 | 8 | 360* | 87 | 27 | 11 | 424* | 121 | 17 | 2 | 249 | 52 | 11 | 3 | 355* | 78 |
| <1 | 273 | 23 | 355 | 75 | 73 | 29 | 418 | 90 | 170 | 17 | 257 | 58 | 86 | 23 | 317 | 71 |
| 1-2 | 619 | 53 | 349 | 80 | 114 | 46 | 397 | 100 | 681 | 68 | 255 | 58 | 238 | 63 | 299 | 68 |
| >2 | 188 | 16 | 342 | 77 | 36 | 14 | 382 | 84 | 137 | 14 | 255 | 63 | 45 | 12 | 302 | 85 |
| Yoghurt, per week | | | | | | | | | | | | | | | | |
| never | 459 | 36 | 345 | 78 | 97 | 36 | 403 | 116 | 265 | 25 | 256 | 60 | 118 | 29 | 311 | 76 |
| <1 | 513 | 41 | 354 | 81 | 114 | 42 | 404 | 87 | 419 | 40 | 253 | 57 | 156 | 39 | 301 | 67 |
| 1-3 | 251 | 20 | 352 | 76 | 50 | 19 | 402 | 83 | 293 | 28 | 261 | 60 | 104 | 26 | 300 | 73 |
| >3 | 41 | 3 | 354 | 71 | 9 | 3 | 363 | 60 | 67 | 6 | 253 | 56 | 23 | 6 | 294 | 56 |
| Milk, glasses per day | | | | | | | | | | | | | | | | |
| never | 59 | 5 | 356* | 91 | 24 | 9 | 408 | 97 | 96 | 10 | 257 | 57 | 36 | 10 | 343*** | 77 |
| <1 | 138 | 12 | 360 | 74 | 23 | 9 | 399 | 100 | 163 | 17 | 262 | 58 | 65 | 18 | 311 | 65 |
| 1-2 | 519 | 44 | 352 | 75 | 95 | 37 | 391 | 73 | 507 | 54 | 252 | 56 | 187 | 52 | 292 | 66 |
| >2 | 456 | 39 | 344 | 83 | 113 | 44 | 405 | 116 | 166 | 18 | 260 | 69 | 69 | 19 | 298 | 69 |
| Cereals or oat meal, times per week | | | | | | | | | | | | | | | | |
| never | 598 | 48 | 350 | 80 | 138 | 51 | 415 | 106 | 439 | 42 | 257 | 58 | 202 | 51 | 307 | 74 |
| <1 | 347 | 28 | 348 | 75 | 68 | 25 | 390 | 95 | 324 | 31 | 254 | 59 | 90 | 23 | 302 | 67 |
| 1 | 127 | 10 | 358 | 84 | 25 | 9 | 388 | 85 | 124 | 12 | 257 | 63 | 48 | 12 | 300 | 64 |
| >1 | 186 | 15 | 356 | 78 | 38 | 14 | 384 | 70 | 153 | 15 | 254 | 57 | 55 | 14 | 294 | 63 |
| Coarse bread, slices per day | | | | | | | | | | | | | | | | |
| <3 | 57 | 4 | 370* | 108 | 18 | 6 | 373 | 52 | 148 | 14 | 263 | 52 | 61 | 15 | 308 | 73 |
| 3-4 | 411 | 32 | 359 | 78 | 119 | 43 | 418 | 97 | 622 | 59 | 257 | 59 | 264 | 65 | 302 | 71 |
| 5-6 | 546 | 43 | 347 | 75 | 105 | 38 | 397 | 107 | 259 | 25 | 247 | 62 | 78 | 19 | 303 | 69 |
| >6 | 261 | 20 | 338 | 77 | 35 | 13 | 380 | 85 | 23 | 2 | 267 | 58 | 5 | 1 | 321 | 68 |
| Fruits (apples, pears, oranges), per week | | | | | | | | | | | | | | | | |
| <1 | 286 | 23 | 352 | 75 | 68 | 25 | 401 | 103 | 170 | 16 | 254 | 59 | 60 | 15 | 322** | 82 |
| 1 | 279 | 22 | 353 | 87 | 57 | 21 | 405 | 96 | 166 | 16 | 251 | 59 | 76 | 19 | 309^a^ | 69 |
| 2-3 | 394 | 31 | 345 | 71 | 86 | 31 | 403 | 94 | 343 | 33 | 255 | 57 | 114 | 28 | 300 | 69 |
| >4 | 307 | 24 | 354 | 84 | 66 | 24 | 402 | 100 | 366 | 35 | 258 | 60 | 157 | 39 | 296 | 68 |

Tromsø Study participants were asked to recall their usual eating habits per week. Intake categories were constructed based on participant responses, to best reflect the patterns of consumption of individual food types as reported by this cohort.

^*^ P-value for linear trend, model adjusted for age decade, BMI (continuous), eGFR (CKD-EPI, continuous), presence of hypertension, presence of diabetes, alcohol intake above 10g/day, use of diuretics, use of anti-gout medication, sweat or dyspnoea-inducing physical activity of 1h and more per week and daily energy intake (kj/day, continuous), *P<0·05, **P<0·01, ***P<0∙001
