# Supplementary material for: Cross-sectional analysis of nutrition and serum uric acid in two Caucasian cohorts: the AusDiab Study and the Tromsø study
Source: Nutr J. 2015 May 14;14:49. doi: 10.1186/s12937-015-0032-1 (PMC4459487; doi:10.1186/s12937-015-0032-1)
Supplement: Additional file 3: Table S3. — Mean serum uric acid (SUA) in males and females, according to intake of alcohol and by presence of central obesity: AusDiab 99/00. [file 12937_2015_32_MOESM3_ESM.docx]

**Supplementary Table 3. Mean serum uric acid (SUA) in males and females, according to intake of alcohol and by presence of central obesity: AusDiab 99/00**

|  | **Males** | | | | | | | | **Females** | | | | | | | |
| --- | --- | --- | --- | --- | --- | --- | --- | --- | --- | --- | --- | --- | --- | --- | --- | --- |
|  | **No abdominal obesity** | | | | **Abdominal obesity** | | | | **No abdominal obesity** | | | | **Abdominal obesity** | | | |
|  | **N** | **%** | **SUA, µmol/l** | | **N** | **%** | **SUA, µmol/l** | | **N** | **%** | **SUA, µmol/l** | | **N** | **%** | **SUA, µmol/l** | |
|  |  |  | **Mean** | **SD** |  |  | **Mean** | **SD** |  |  | **Mean** | **SD** |  |  | **Mean** | **SD** |
| Alcohol, g per day | | | | | | | | | | | | | | | | |
| 0 | 260 | 9 | 319*** | 72 | 134 | 10 | 355*** | 73 | 531 | 15 | 240*** | 66 | 425 | 21 | 303* | 81 |
| <5 | 644 | 22 | 320 | 70 | 345 | 25 | 363 | 70 | 1383 | 40 | 227 | 58 | 873 | 44 | 286 | 73 |
| 5-10 | 346 | 12 | 326 | 67 | 182 | 13 | 375 | 73 | 473 | 14 | 227 | 58 | 224 | 11 | 284 | 70 |
| >10 | 1644 | 57 | 337 | 73 | 740 | 53 | 378 | 80 | 1065 | 31 | 236 | 61 | 465 | 23 | 290 | 75 |
| Beer (full-strength), days per week | | | | | | | | | | | | | | | | |
| 0 | 887 | 31 | 321*** | 71 | 531 | 38 | 363*** | 75 | 2663 | 77 | 232*** | 61 | 1634 | 82 | 290*** | 76 |
| <1 | 974 | 34 | 327 | 72 | 431 | 31 | 368 | 76 | 577 | 17 | 225 | 56 | 287 | 14 | 291 | 72 |
| 1-2 | 520 | 18 | 330 | 67 | 209 | 15 | 380 | 76 | 137 | 4 | 231 | 57 | 38 | 2 | 286 | 59 |
| >=3 | 513 | 18 | 353 | 76 | 230 | 16 | 392 | 77 | 75 | 2 | 262 | 67 | 28 | 1 | 337 | 88 |
| Wine (red or white), per week | | | | | | | | | | | | | | | | |
| 0 | 735 | 25 | 331* | 75 | 408 | 29 | 371 | 78 | 789 | 23 | 240 | 65 | 650 | 33 | 301 | 78 |
| <1 | 870 | 30 | 335 | 74 | 403 | 29 | 377 | 74 | 1099 | 32 | 229 | 60 | 675 | 34 | 286 | 75 |
| 1-2 | 574 | 20 | 326 | 71 | 267 | 19 | 373 | 80 | 668 | 19 | 226 | 56 | 282 | 14 | 284 | 67 |
| >=3 | 715 | 25 | 328 | 69 | 323 | 23 | 366 | 74 | 896 | 26 | 232 | 60 | 380 | 19 | 285 | 74 |
| Spirits, days per week | | | | | | | | | | | | | | | | |
| 0 | 1120 | 39 | 325* | 73 | 582 | 42 | 367 | 73 | 1649 | 48 | 235* | 64 | 1034 | 52 | 293 | 78 |
| <1 | 1320 | 46 | 331 | 70 | 613 | 44 | 373 | 75 | 1390 | 40 | 227 | 55 | 770 | 39 | 287 | 73 |
| 1-2 | 291 | 10 | 334 | 77 | 129 | 9 | 386 | 87 | 244 | 7 | 229 | 59 | 99 | 5 | 287 | 61 |
| >=3 | 163 | 6 | 351 | 78 | 77 | 5 | 378 | 87 | 169 | 5 | 246 | 69 | 84 | 4 | 295 | 65 |

AusDiab participants were asked to recall their alcohol consumption habits averaged over the past 12 months. Intake categories were constructed based on participant responses, to best reflect the patterns of consumption as reported by this cohort.

^*^ P-value for linear trend, model adjusted for age (continuous), BMI (continuous), eGFR (CKD-EPI, continuous), presence of hypertension, presence of diabetes, alcohol intake above 10g/day, self-reported history of gout at baseline, 1h or more of vigorous physical activity in the past week, energy intake (kj/day, continuous) *P<0•05, **P<0•01, ***P<0∙001
