# Supplementary material for: Cross-sectional analysis of nutrition and serum uric acid in two Caucasian cohorts: the AusDiab Study and the Tromsø study
Source: Nutr J. 2015 May 14;14:49. doi: 10.1186/s12937-015-0032-1 (PMC4459487; doi:10.1186/s12937-015-0032-1)
Supplement: Additional file 5: Table S5. — “Serum Uric Acid in Gender- and Obesity Group-specific quartiles of Nutrient Intake. The AusDiab 1999/00”. [file 12937_2015_32_MOESM5_ESM.docx]

**Supplementary Table 5. Serum Uric Acid in Gender- and Obesity Group-specific quartiles of Nutrient Intake. The AusDiab 1999/2000.**

|  |  | **Males** | | | | | | **Females** | | | | | |
| --- | --- | --- | --- | --- | --- | --- | --- | --- | --- | --- | --- | --- | --- |
|  |  | **No abdominal obesity** | | | **Abdominal obesity** | | | **No abdominal obesity** | | | **Abdominal obesity** | | |
|  |  |  | **SUA, µmol/l** | |  | **SUA, µmol/l** | |  | **SUA, µmol/l** | |  | **SUA, µmol/l** | |
| **Daily intake** | **Q**^1^ | ***Upper cutoff*** | **Unadj^2^** | **Adj^3^** | ***Upper cutoff*** | **Unadj** | **Adj** | ***Upper cutoff*** | **Unadj** | **Adj** | ***Upper cutoff*** | **Unadj** | **Adj** |
| Energy, | 1 | *7170* | 334 | 334 | *7019* | 379 | 381 | *5366* | 235 | 234 | *5459* | 292 | 290 |
| Kj | 2 | *8940* | 334 | 333 | *8694* | 367 | 367 | *6637* | 235 | 233 | *6884* | 287 | 288 |
|  | 3 | *11306* | 326 | 326 | *11085* | 367 | 368 | *8276* | 229 | 230 | *8545* | 289 | 290 |
|  | 4 | *37891* | 328 | 329 | *52919* | 375 | 372 | *50773* | 229 | 232 | *58009* | 294 | 295 |
| **Linear trend, *p*** |  |  | **0.047** | **0.07** |  | **0.48** | **0.13** |  | **0.004** | **0.36** |  | **0.67** | **0.25** |
| Protein, | 1 | *79* | 334 | 331 | *79* | 376 | 380 | *61* | 235 | 233 | *63* | 292 | 291 |
| g | 2 | *100* | 330 | 328 | *100* | 369 | 370 | *76* | 233 | 231 | *80* | 288 | 290 |
|  | 3 | *127* | 325 | 324 | *130* | 368 | 367 | *94* | 229 | 231 | *101* | 289 | 291 |
|  | 4 | *557* | 333 | 338 | *1075* | 374 | 372 | *995* | 230 | 234 | *683* | 292 | 290 |
| **Linear trend, *p*** |  |  | **0.61** | **0.37** |  | **0.78** | **0.30** |  | **0.052** | **0.89** |  | **0.98** | **0.97** |
| Protein | 1 | *17* | 322 | 326 | *18* | 374 | 378 | *18* | 229 | 231 | *18* | 293 | 292 |
| Kj% | 2 | *19* | 333 | 332 | *20* | 366 | 366 | *19* | 231 | 231 | *20* | 291 | 290 |
|  | 3 | *21* | 329 | 330 | *22* | 372 | 371 | *21* | 230 | 229 | *22* | 288 | 290 |
|  | 4 | *33* | 337 | 334 | *37* | 376 | 374 | *37* | 238 | 237 | *42* | 290 | 291 |
| **Linear trend*, p*** |  |  | **0.001** | **0.09** |  | **0.57** | **0.54** |  | **0.007** | **0.06** |  | **0.34** | **0.85** |
| Carbohydrate, | 1 | *188* | 339 | 343 | *178* | 384 | 392 | *145* | 238 | 239 | *143* | 300 | 304 |
| g | 2 | *236* | 331 | 332 | *227* | 372 | 376 | *180* | 234 | 235 | *182* | 285 | 286 |
|  | 3 | *298* | 328 | 328 | *281* | 363 | 363 | *224* | 230 | 229 | *228* | 288 | 288 |
|  | 4 | *1224* | 323 | 319 | *753* | 368 | 358 | *704* | 225 | 225 | *1436* | 289 | 284 |
| **Linear trend, *p*** |  |  | **0.000** | **0.000** |  | **0.002** | **0.000** |  | **0.000** | **0.000** |  | **0.06** | **0.002** |
| Carbohydrate, | 1 | *41* | 341 | 340 | *40* | 384 | 383 | *42* | 238 | 242 | *41* | 301 | 302 |
| Kj% | 2 | *45* | 331 | 332 | *44* | 380 | 376 | *46* | 231 | 232 | *45* | 294 | 294 |
|  | 3 | *49* | 330 | 329 | *48* | 366 | 368 | *50* | 232 | 230 | *49* | 287 | 286 |
|  | 4 | *72* | 319 | 321 | *69* | 357 | 361 | *69* | 226 | 225 | *75* | 281 | 281 |
| **Linear trend, *p*** |  |  | **0.000** | **0.000** |  | **0.000** | **0.000** |  | **0.000** | **0.000** |  | **0.000** | **0.000** |
| Sugar, | 1 | *74* | 338 | 341 | *69* | 386 | 387 | *62* | 237 | 240 | *61* | 298 | 303 |
| g | 2 | *99* | 334 | 334 | *92* | 366 | 370 | *80* | 233 | 234 | *80* | 289 | 289 |
|  | 3 | *128* | 330 | 329 | *121* | 369 | 371 | *102* | 230 | 230 | *103* | 290 | 289 |
|  | 4 | *357* | 320 | 318 | *406* | 367 | 361 | *343* | 227 | 225 | *456* | 286 | 282 |
| **Linear trend, *p*** |  |  | **0.000** | **0.000** |  | **0.002** | **0.000** |  | **0.000** | **0.000** |  | **0.023** | **0.000** |
| Fiber, | 1 | *18* | 340 | 342 | *18* | 387 | 387 | *15* | 235 | 238 | *16* | 299 | 304 |
| g | 2 | *24* | 335 | 334 | *23* | 373 | 376 | *20* | 236 | 237 | *20* | 291 | 292 |
|  | 3 | *31* | 324 | 324 | *30* | 363 | 365 | *25* | 230 | 230 | *25* | 287 | 288 |
|  | 4 | *117* | 323 | 322 | *81* | 364 | 361 | *68* | 226 | 223 | *117* | 285 | 280 |
| **Linear trend, *p*** |  |  | **0.000** | **0.000** |  | **0.000** | **0.000** |  | **0.000** | **0.000** |  | **0.002** | **0.000** |
| Total fat, | 1 | *68* | 330 | 324 | *67* | 371 | 368 | *47* | 234 | 230 | *50* | 287 | 285 |
| g | 2 | *89* | 334 | 329 | *87* | 367 | 367 | *62* | 234 | 230 | *65* | 289 | 286 |
|  | 3 | *115* | 326 | 328 | *113* | 373 | 373 | *81* | 230 | 232 | *84* | 292 | 294 |
|  | 4 | *453* | 331 | 341 | *663* | 377 | 381 | *654* | 23 | 237 | *627* | 295 | 297 |
| **Linear trend, *p*** |  |  | **0.82** | **0.012** |  | **0.18** | **0.09** |  | **0.09** | **0.058** |  | **0.07** | **0.021** |
| Total fat, | 1 | *33* | 324 | 323 | *34* | 352 | 355 | *31* | 226 | 224 | *32* | 278 | 278 |
| Kj% | 2 | *37* | 328 | 328 | *38* | 375 | 376 | *35* | 231 | 229 | *36* | 287 | 287 |
|  | 3 | *40* | 330 | 330 | *41* | 375 | 375 | *39* | 237 | 238 | *39* | 295 | 293 |
|  | 4 | *57* | 340 | 341 | *51* | 385 | 383 | *60* | 233 | 238 | *52* | 303 | 304 |
| **Linear trend, *p*** |  |  | **0.000** | **0.000** |  | **0.000** | **0.000** |  | **0.003** | **0.000** |  | **0.000** | **0.000** |
| SFA, | 1 | *26* | 329 | 326 | *26* | 363 | 361 | *18* | 234 | 229 | *19* | 286 | 284 |
| g | 2 | *36* | 333 | 329 | *34* | 377 | 373 | *25* | 235 | 230 | *25* | 290 | 289 |
|  | 3 | *48* | 329 | 330 | *47* | 368 | 370 | *33* | 228 | 230 | *34* | 292 | 292 |
|  | 4 | *185* | 330 | 337 | *273* | 379 | 384 | *266* | 231 | 239 | *275* | 294 | 298 |
| **Linear trend, *p*** |  |  | **0.89** | **0.046** |  | **0.039** | **0.010** |  | **0.08** | **0.009** |  | **0.06** | **0.015** |
| MUFA, | 1 | *10* | 330 | 324 | *10* | 368 | 369 | *7* | 236 | 230 | *8* | 288 | 286 |
| g | 2 | *14* | 333 | 328 | *14* | 371 | 370 | *10* | 231 | 227 | *11* | 289 | 287 |
|  | 3 | *19* | 326 | 327 | *18* | 373 | 373 | *14* | 229 | 232 | *15* | 292 | 294 |
|  | 4 | *50* | 332 | 343 | *63* | 376 | 376 | *65* | 231 | 239 | *71* | 293 | 296 |
| **Linear trend, *p*** |  |  | **0.98** | **0.004** |  | **0.18** | **0.35** |  | **0.07** | **0.017** |  | **0.20** | **0.057** |
| PUFA, | 1 | *23* | 332 | 331 | *23* | 371 | 370 | *16* | 232 | 233 | *17* | 287 | 290 |
| g | 2 | *31* | 333 | 333 | *31* | 370 | 372 | *22* | 234 | 233 | *23* | 293 | 294 |
|  | 3 | *40* | 328 | 327 | *40* | 372 | 373 | *29* | 230 | 230 | *30* | 290 | 288 |
|  | 4 | *177* | 328 | 331 | *264* | 375 | 374 | *265* | 230 | 232 | *228* | 292 | 291 |
| **Linear trend, *p*** |  |  | **0.20** | **0.77** |  | **0.48** | **0.55** |  | **0.27** | **0.59** |  | **0.36** | **0.85** |
| Cholesterol, | 1 | *225* | 326 | 322 | *228* | 373 | 373 | *168* | 232 | 227 | *176* | 286 | 282 |
| mg | 2 | *303* | 329 | 325 | *310* | 363 | 363 | *220* | 231 | 227 | *234* | 290 | 288 |
|  | 3 | *406* | 329 | 330 | *421* | 376 | 374 | *291* | 232 | 235 | *303* | 287 | 289 |
|  | 4 | *1880* | 337 | 345 | *3683* | 376 | 378 | *3331* | 232 | 240 | *2262* | 300 | 303 |
| **Linear trend, *p*** |  |  | **0.012** | **0.000** |  | **0.21** | **0.31** |  | **0.98** | **0.000** |  | **0.009** | **0.000** |
| Retinol, | 1 | *0,3* | 332 | 328 | *0,3* | 367 | 365 | *0,2* | 231 | 230 | *0,2* | 286 | 288 |
| mg | 2 | *0,4* | 334 | 333 | *0,4* | 371 | 374 | *0,3* | 234 | 231 | *0,3* | 292 | 292 |
|  | 3 | *0,6* | 331 | 332 | *0,6* | 376 | 375 | *0,4* | 231 | 231 | *0,4* | 286 | 283 |
|  | 4 | *1,7* | 324 | 329 | *1,3* | 373 | 374 | *1,5* | 231 | 237 | *2,4* | 298 | 301 |
| **Linear trend, *p*** |  |  | **0.030** | **0.97** |  | **0.20** | **0.17** |  | **0.78** | **0.048** |  | **0.048** | **0.06** |
| Retinol eq, | 1 | *0,7* | 3312 | 328 | *0,7* | 374 | 372 | *0,6* | 232 | 231 | *0,6* | 291 | 292 |
| mg | 2 | *0,9* | 335 | 333 | *0,9* | 372 | 374 | *0,7* | 237 | 235 | *0,8* | 287 | 287 |
|  | 3 | *1,2* | 328 | 328 | *1,1* | 366 | 367 | *0,9* | 229 | 230 | *1,0* | 292 | 293 |
|  | 4 | *3,1* | 328 | 332 | *2,8* | 375 | 375 | *3,9* | 230 | 233 | *3,7* | 292 | 292 |
| **Linear trend, *p*** |  |  | **0.16** | **0.60** |  | **0.84** | **0.93** |  | **0.16** | **0.89** |  | **0.59** | **0.73** |
| Β-carotene, | 1 | *1,8* | 337 | 337 | *1,8* | 376 | 375 | *1,6* | 231 | 232 | *1,7* | 295 | 294 |
| mg | 2 | *2,6* | 325 | 324 | *2,6* | 370 | 371 | *2,3* | 235 | 235 | *2,4* | 288 | 289 |
|  | 3 | *3,6* | 329 | 329 | *3,6* | 371 | 370 | *3,1* | 233 | 232 | *3,3* | 289 | 289 |
|  | 4 | *14,8* | 330 | 331 | *15,1* | 372 | 373 | *19,3* | 228 | 230 | *10,6* | 290 | 290 |
| **Linear trend, *p*** |  |  | **0.24** | **0.40** |  | **0.51** | **0.81** |  | **0.26** | **0.31** |  | **0.38** | **0.41** |
| Vitamin C, | 1 | *80* | 335 | 335 | *78* | 380 | 379 | *73* | 235 | 237 | *74* | 297 | 300 |
| mg | 2 | *116* | 329 | 329 | *119* | 369 | 369 | *106* | 233 | 233 | *106* | 290 | 288 |
|  | 3 | *171* | 331 | 330 | *175* | 370 | 373 | *148* | 232 | 230 | *152* | 282 | 284 |
|  | 4 | *807* | 326 | 328 | *811* | 368 | 368 | *934* | 227 | 228 | *706* | 293 | 291 |
| **Linear trend, *p*** |  |  | **0.045** | **0.11** |  | **0.043** | **0.13** |  | **0.003** | **0.004** |  | **0.19** | **0.043** |
| Vitamin E, | 1 | *5,5* | 334 | 335 | *5,4* | 380 | 381 | *4,2* | 236 | 238 | *4,3* | 291 | 292 |
| mg | 2 | *7,2* | 335 | 333 | *7,0* | 368 | 371 | *5,4* | 235 | 234 | *5,6* | 293 | 296 |
|  | 3 | *9,2* | 329 | 329 | *8,9* | 368 | 368 | *7,0* | 229 | 229 | *7,0* | 287 | 288 |
|  | 4 | *25,5* | 323 | 324 | *24,6* | 371 | 369 | *22,3* | 226 | 226 | *36,7* | 290 | 288 |
| **Linear trend, *p*** |  |  | **0.002** | **0.024** |  | **0.16** | **0.12** |  | **0.000** | **0.001** |  | **0.59** | **0.22** |
| Calcium, | 1 | *710* | 343 | 346 | *676* | 379 | 383 | *650* | 242 | 244 | *660* | 300 | 304 |
| mg | 2 | *904* | 335 | 334 | *884* | 377 | 378 | *845* | 230 | 232 | *852* | 290 | 290 |
|  | 3 | *1148* | 327 | 326 | *1111* | 368 | 369 | *1061* | 229 | 229 | *1066* | 288 | 289 |
|  | 4 | *2643* | 317 | 316 | *3000* | 364 | 359 | *2504* | 226 | 224 | *5004* | 284 | 280 |
| **Linear trend, *p*** |  |  | **0.000** | **0.000** |  | **0.004** | **0.000** |  | **0.000** | **0.000** |  | **0.001** | **0.000** |
| Iron, | 1 | *11,2* | 338 | 340 | *10,9* | 384 | 390 | *8,3* | 236 | 238 | *8,7* | 294 | 296 |
| mg | 2 | *14,6* | 330 | 330 | *14,2* | 371 | 374 | *10,9* | 235 | 235 | *11,2* | 293 | 294 |
|  | 3 | *19,0* | 328 | 327 | *18,6* | 365 | 365 | *14,1* | 229 | 229 | *14,4* | 286 | 286 |
|  | 4 | *65,9* | 326 | 324 | *95,8* | 368 | 360 | *97,5* | 227 | 226 | *102,1* | 290 | 287 |
| **Linear trend, *p*** |  |  | **0.002** | **0.003** |  | **0.002** | **0.000** |  | **0.000** | **0.001** |  | **0.21** | **0.050** |
| Vitamin B1, | 1 | *1,36* | 338 | 340 | *1,33* | 381 | 384 | *1,05* | 236 | 238 | *1,11* | 295 | 299 |
| mg | 2 | *1,78* | 332 | 332 | *1,73* | 369 | 371 | *1,37* | 234 | 233 | *1,45* | 285 | 285 |
|  | 3 | *2,40* | 327 | 327 | *2,27* | 368 | 370 | *1,81* | 230 | 230 | *1,88* | 292 | 291 |
|  | 4 | *9,70* | 324 | 322 | *10,37* | 369 | 364 | *11,48* | 227 | 227 | *11,73* | 290 | 288 |
| **Linear trend, *p*** |  |  | **0.000** | **0.000** |  | **0.051** | **0.009** |  | **0.001** | **0.002** |  | **0.68** | **0.15** |
| Vitamin B2, | 1 | *1,89* | 339 | 341 | *1,82* | 381 | 386 | *1,60* | 237 | 239 | *1,64* | 298 | 300 |
| mg | 2 | *2,55* | 330 | 331 | *2,46* | 372 | 374 | *2,12* | 234 | 234 | *2,20* | 289 | 291 |
|  | 3 | *3,41* | 329 | 329 | *3,31* | 366 | 368 | *2,77* | 228 | 229 | *2,88* | 285 | 284 |
|  | 4 | *12,96* | 323 | 321 | *13,74* | 368 | 362 | *15,58* | 228 | 226 | *16,34* | 290 | 288 |
| **Linear trend, *p*** |  |  | **0.000** | **0.000** |  | **0.014** | **0.000** |  | **0.000** | **0.000** |  | **0.07** | **0.006** |
| Folate, | 1 | *226* | 338 | 340 | *224* | 384 | 386 | *186* | 237 | 241 | *193* | 293 | 297 |
| mg | 2 | *293* | 334 | 333 | *288* | 370 | 373 | *241* | 235 | 234 | *247* | 294 | 294 |
|  | 3 | *383* | 325 | 326 | *368* | 367 | 367 | *310* | 229 | 229 | *314* | 288 | 289 |
|  | 4 | *1333* | 325 | 323 | *1049* | 367 | 363 | *1073* | 226 | 224 | *1564* | 287 | 283 |
| **Linear trend, *p*** |  |  | **0.000** | **0.000** |  | **0.003** | **0.001** |  | **0.000** | **0.000** |  | **0.10** | **0.006** |
| Zinc, | 1 | *10* | 334 | 333 | *10* | 378 | 382 | *8* | 233 | 231 | *8* | 290 | 288 |
| mg | 2 | *13* | 328 | 326 | *13* | 370 | 370 | *10* | 233 | 231 | *10* | 286 | 286 |
|  | 3 | *17* | 326 | 325 | *17* | 364 | 365 | *12* | 230 | 232 | *13* | 292 | 294 |
|  | 4 | *75* | 333 | 338 | *145* | 376 | 372 | *136* | 231 | 234 | *102* | 295 | 295 |
| **Linear trend, *p*** |  |  | **0.67** | **0.41** |  | **0.57** | **0.16** |  | **0.30** | **0.41** |  | **0.18** | **0.17** |

^1^ Q, quartile of intake, ^2^ Unadj, unadjasted values, ^3^ adj, multiple adjustment for age, BMI, eGFR (SKD-EPI), dietary energy intake kJ/day (except for Energy quartiles), alcohol intake >10g/day, physical activity, diabetes, hypertension, gout
