# Supplementary material for: Cross-sectional analysis of nutrition and serum uric acid in two Caucasian cohorts: the AusDiab Study and the Tromsø study
Source: Nutr J. 2015 May 14;14:49. doi: 10.1186/s12937-015-0032-1 (PMC4459487; doi:10.1186/s12937-015-0032-1)
Supplement: Additional file 6: Table S6. — “Serum Uric Acid in Gender- and Obesity Status-specific quartiles of Nutrient Intake. The Tromsø Study 1994/95’. [file 12937_2015_32_MOESM6_ESM.docx]

**Supplementary Table 6. Serum Uric Acid in Gender- and Obesity Status-specific quartiles of Nutrient Intake.The Tromsø Study 1994/95.**

|  |  | **Males** | | | | | | **Females** | | | | | |
| --- | --- | --- | --- | --- | --- | --- | --- | --- | --- | --- | --- | --- | --- |
|  |  | **No abdominal obesity** | | | **Abdominal obesity** | | | **No abdominal obesity** | | | **Abdominal obesity** | | |
|  |  |  | **SUA, µmol/l** | |  | **SUA, µmol/l** | |  | **SUA, µmol/l** | |  | **SUA, µmol/l** | |
|  | Q^1^ | ***Upper cutoff*** | **Unadj^2^** | **Adj^3^** | ***Upper cutoff*** | **Unadj** | **Adj** | ***Upper cutoff*** | **Unadj** | **Adj** | ***Upper cutoff*** | **Unadj** | **Adj** |
| Energy, | 1 | *7713* | 352 | 351 | *7503* | 402 | 401 | *5567* | 260 | 259 | *5254* | 303 | 297 |
| Kj | 2 | *9051* | 351 | 350 | *8677* | 421 | 422 | *6687* | 256 | 253 | *6356* | 311 | 306 |
|  | 3 | *10310* | 355 | 355 | *10000* | 381 | 383 | *7783* | 251 | 254 | *7438* | 301 | 306 |
|  | 4 | *14958* | 342 | 346 | *15104* | 405 | 402 | *11634* | 256 | 257 | *11369* | 299 | 305 |
| **Linear trend, *p*** |  |  | **0.18** | **0.67** |  | **0.54** | **0.49** |  | **0.33** | **0.75** |  | **0.46** | **0.42** |
| Protein, | 1 | *77* | 350 | 353 | *80* | 402 | 405 | *57* | 259 | 263 | *56* | 306 | 305 |
| g | 2 | *88* | 356 | 353 | *89* | 411 | 406 | *66* | 256 | 258 | *67* | 303 | 301 |
|  | 3 | *101* | 349 | 349 | *103* | 401 | 405 | *76* | 253 | 253 | *76* | 300 | 301 |
|  | 4 | *228* | 347 | 346 | *148* | 395 | 392 | *122* | 254 | 248 | *123* | 305 | 306 |
| **Linear trend, *p*** |  |  | **0.44** | **0.37** |  | **0.55** | **0.59** |  | **0.27** | **0.047** |  | **0.87** | **0.90** |
| Protein, | 1 | *15* | 345 | 351 | *16* | 402 | 399 | *16* | 253 | 256 | *16* | 305 | 306 |
| Kj% | 2 | *17* | 348 | 350 | *18* | 390 | 397 | *17* | 255 | 256 | *18* | 295 | 298 |
|  | 3 | *18* | 358 | 355 | *19* | 424 | 424 | *18* | 257 | 256 | *19* | 299 | 298 |
|  | 4 | *29* | 350 | 345 | *25* | 393 | 387 | *27* | 258 | 255 | *27* | 315 | 311 |
| **Linear trend, *p*** |  |  | **0.18** | **0.49** |  | **0.89** | **0.88** |  | **0.33** | **0.96** |  | **0.28** | **0.61** |
| Carbohydrate, | 1 | *238* | 353 | 346 | *237* | 409 | 415 | *164* | 261 | 266 | *156* | 315 | 321 |
| g | 2 | *286* | 354 | 351 | *269* | 412 | 419 | *203* | 250 | 252 | *193* | 303 | 311 |
|  | 3 | *329* | 342 | 343 | *317* | 399 | 389 | *242* | 256 | 255 | *233* | 298 | 295 |
|  | 4 | *492* | 352 | 361 | *488* | 388 | 386 | *394* | 256 | 249 | *376* | 298 | 286 |
| **Linear trend, *p*** |  |  | **0.46** | **0.23** |  | **0.15** | **0.15** |  | **0.50** | **0.08** |  | **0.08** | **0.033** |
| Carbohydrate, | 1 | *50* | 351 | 351 | *49* | 406 | 406 | *48* | 255 | 259 | *48* | 312 | 308 |
| Kj% | 2 | *54* | 352 | 351 | *53* | 407 | 402 | *52* | 259 | 259 | *52* | 304 | 306 |
|  | 3 | *58* | 348 | 349 | *57* | 391 | 387 | *56* | 252 | 252 | *55* | 303 | 300 |
|  | 4 | *72* | 350 | 351 | *69* | 405 | 413 | *79* | 257 | 253 | *72* | 295 | 299 |
| **Linear trend, *p*** |  |  | **0.69** | **0.85** |  | **0.72** | **0.90** |  | **0.95** | **0.10** |  | **0.10** | **0.26** |
| Sugar, | 1 | *25* | 358 | 354 | *20* | 404 | 403 | *20* | 258 | 253 | *16* | 310 | 305 |
| g | 2 | *36* | 344 | 343 | *30* | 410 | 414 | *31* | 257 | 258 | *27* | 297 | 301 |
|  | 3 | *54* | 351 | 352 | *43* | 398 | 395 | *43* | 253 | 254 | *37* | 304 | 302 |
|  | 4 | *175* | 348 | 353 | *127* | 396 | 395 | *163* | 255 | 257 | *90* | 303 | 305 |
| **Linear trend, *p*** |  |  | **0.18** | **0.77** |  | **0.49** | **0.43** |  | **0.47** | **0.71** |  | **0.63** | **0.97** |
| Fiber, | 1 | *22* | 351 | 349 | *21* | 415 | 406 | *15* | 259 | 263 | *15* | 306 | 304 |
| g | 2 | *26* | 352 | 351 | *25* | 408 | 405 | *19* | 253 | 255 | *19* | 303 | 304 |
|  | 3 | *31* | 349 | 348 | *30* | 383 | 387 | *23* | 254 | 252 | *22* | 306 | 304 |
|  | 4 | *47* | 350 | 353 | *47* | 402 | 410 | *37* | 257 | 252 | *33* | 299 | 300 |
| **Linear trend, *p*** |  |  | **0.69** | **0.76** |  | **0.22** | **0.90** |  | **0.72** | **0.07** |  | **0.56** | **0.77** |
| Total fat, | 1 | *54* | 355 | 354 | *52* | 403 | 402 | *42* | 260 | 255 | *40* | 300 | 295 |
| g | 2 | *68* | 356 | 351 | *64* | 410 | 407 | *55* | 252 | 250 | *50* | 302 | 297 |
|  | 3 | *83* | 342 | 345 | *81* | 402 | 409 | *68* | 257 | 259 | *63* | 307 | 309 |
|  | 4 | *154* | 348 | 352 | *144* | 392 | 390 | *132* | 253 | 258 | *124* | 305 | 312 |
| **Linear trend, *p*** |  |  | **0.07** | **0.64** |  | **0.44** | **0.64** |  | **0.38** | **0.44** |  | **0.49** | **0.17** |
| Totalfat, | 1 | *24* | 352 | 351 | *24* | 401 | 400 | *27* | 257 | 253 | *26* | 295 | 295 |
| Kj% | 2 | *28* | 350 | 348 | *28* | 416 | 413 | *31* | 254 | 253 | *30* | 299 | 300 |
|  | 3 | *32* | 352 | 352 | *31* | 383 | 388 | *34* | 256 | 256 | *34* | 310 | 309 |
|  | 4 | *49* | 347 | 350 | *43* | 409 | 408 | *48* | 255 | 262 | *48* | 311 | 309 |
| **Linear trend, *p*** |  |  | **0.43** | **0.93** |  | **0.87** | **0.99** |  | **0.85** | **0.053** |  | **0.06** | **0.09** |
| SFA, | 1 | *22* | 358 | 356 | *20* | 399 | 407 | *18* | 259 | 255 | *17* | 301 | 304 |
| g | 2 | *28* | 351 | 350 | *26* | 435 | 429 | *24* | 255 | 255 | *21* | 312 | 308 |
|  | 3 | *35* | 346 | 346 | *32* | 385 | 389 | *29* | 255 | 257 | *27* | 297 | 299 |
|  | 4 | *69* | 346 | 350 | *72* | 389 | 383 | *61* | 253 | 256 | *61* | 305 | 302 |
| **Linear trend, *p*** |  |  | **0.034** | **0.37** |  | **0.13** | **0.13** |  | **0.23** | **0.84** |  | **0.90** | **0.70** |
| MUFA, | 1 | *18* | 353 | 351 | *17* | 404 | 412 | *14* | 259 | 253 | *13* | 300 | 294 |
| g | 2 | *22* | 356 | 351 | *21* | 413 | 413 | *18* | 252 | 250 | *16* | 297 | 293 |
|  | 3 | *27* | 341 | 344 | *26* | 406 | 408 | *22* | 259 | 260 | *20* | 309 | 309 |
|  | 4 | *58* | 351 | 355 | *48* | 385 | 375 | *41* | 253 | 259 | *39* | 309 | 317 |
| **Linear trend, *p*** |  |  | **0.27** | **0.85** |  | **0.22** | **0.10** |  | **0.50** | **0.28** |  | **0.21** | **0.051** |
| PUFA, | 1 | *8* | 350 | 347 | *7* | 393 | 386 | *6* | 259 | 257 | *6* | 293 | 287 |
| g | 2 | *11* | 357 | 356 | *10* | 403 | 403 | *8* | 257 | 254 | *8* | 303 | 301 |
|  | 3 | *15* | 353 | 350 | *14* | 407 | 407 | *11* | 251 | 251 | *10* | 311 | 313 |
|  | 4 | *35* | 342 | 348 | *31* | 406 | 411 | *27* | 257 | 260 | *25* | 308 | 313 |
| **Linear trend, *p*** |  |  | **0.20** | **0.92** |  | **0.41** | **0.20** |  | **0.47** | **0.73** |  | **0.08** | **0.012** |
| Omega3, | 1 | *0.5* | 345 | 348 | *0.5* | 384 | 386 | *0.2* | 256 | 256 | *0.4* | 296 | 290 |
| g | 2 | *0.8* | 349 | 348 | *0.9* | 413 | 411 | *0.6* | 253 | 256 | *0.6* | 300 | 305 |
|  | 3 | *1.6* | 355 | 354 | *1.6* | 407 | 409 | *0.8* | 254 | 254 | *0.8* | 304 | 305 |
|  | 4 | *4.9* | 352 | 352 | *6.1* | 404 | 402 | *4.3* | 259 | 256 | *3.7* | 316 | 313 |
| **Linear trend, *p*** |  |  | **0.18** | **0.38** |  | **0.31** | **0.38** |  | **0.51** | **0.98** |  | **0.040** | **0.017** |
| Cholesterol, | 1 | *211* | 350 | 348 | *216* | 409 | 410 | *168* | 257 | 255 | *168* | 303 | 300 |
| mg | 2 | *257* | 347 | 345 | *261* | 395 | 391 | *209* | 250 | 252 | *206* | 295 | 295 |
|  | 3 | *308* | 351 | 350 | *318* | 407 | 407 | *254* | 256 | 255 | *257* | 312 | 311 |
|  | 4 | *729* | 353 | 357 | *583* | 398 | 401 | *572* | 260 | 260 | *473* | 304 | 306 |
| **Linear trend, *p*** |  |  | **0.45** | **0.18** |  | **0.69** | **0.84** |  | **0.40** | **0.38** |  | **0.47** | **0.38** |
| Retinol, | 1 | *0.8* | 355 | 353 | *0.8* | 412 | 406 | *0.9* | 255 | 257 | *0.9* | 297 | 301 |
| mg | 2 | *1.1* | 350 | 349 | *1.0* | 403 | 398 | *1.2* | 256 | 256 | *1.2* | 300 | 300 |
|  | 3 | *1.4* | 345 | 348 | *1.4* | 382 | 388 | *1.5* | 252 | 250 | *1.5* | 318 | 316 |
|  | 4 | *2.6* | 351 | 351 | *2.4* | 411 | 417 | *2.6* | 259 | 259 | *2.5* | 299 | 296 |
| **Linear trend, *p*** |  |  | **0.40** | **0.70** |  | **0.61** | **0.68** |  | **0.62** | **0.96** |  | **0.42** | **0.99** |
| Β-Carotene, | 1 | *2.5* | 354 | 353 | *2.5* | 412 | 407 | *3.3* | 256 | 257 | *3.3* | 292 | 297 |
| mg | 2 | *3.5* | 349 | 351 | *3.5* | 385 | 389 | *4.5* | 251 | 253 | *5.5* | 303 | 301 |
|  | 3 | *6.5* | 347 | 348 | *6.0* | 400 | 394 | *6.6* | 254 | 252 | *6.6* | 312 | 311 |
|  | 4 | *9.7* | 351 | 349 | *9.5* | 410 | 417 | *9.4* | 261 | 260 | *9.5* | 307 | 303 |
| **Linear trend, *p*** |  |  | **0.54** | **0.38** |  | **0.87** | **0.49** |  | **0.33** | **0.66** |  | **0.07** | **0.34** |
| Vitamin C, | 1 | *95* | 346 | 346 | *96* | 401 | 398 | *68* | 252 | 253 | *72* | 309 | 305 |
| mg | 2 | *124* | 357 | 355 | *126* | 389 | 392 | *97* | 257 | 257 | *99* | 306 | 309 |
|  | 3 | *160* | 348 | 348 | *161* | 394 | 395 | *130* | 257 | 256 | *131* | 300 | 300 |
|  | 4 | *461* | 351 | 352 | *400* | 424 | 424 | *331* | 257 | 256 | *365* | 299 | 299 |
| **Linear trend, *p*** |  |  | **0.80** | **0.61** |  | **0.14** | **0.13** |  | **0.38** | **0.74** |  | **0.25** | **0.41** |
| Vitamin D, | 1 | *3* | 348 | 347 | *2* | 403 | 397 | *2* | 254 | 254 | *2* | 287 | 288 |
| mg | 2 | *4* | 350 | 350 | *4* | 392 | 393 | *3* | 256 | 257 | *3* | 313 | 307 |
|  | 3 | *6* | 356 | 356 | *7* | 413 | 416 | *4* | 256 | 256 | *5* | 299 | 301 |
|  | 4 | *24* | 348 | 350 | *22* | 401 | 403 | *16* | 258 | 255 | *15* | 323 | 322 |
| **Linear trend, *p*** |  |  | **0.68** | **0.45** |  | **0.79** | **0.49** |  | **0.40** | **0.91** |  | **0.007** | **0.003** |
| Vitamin E, | 1 | *5.4* | 348 | 344 | *5.6* | 405 | 388 | *4.0* | 253 | 258 | *3.9* | 297 | 300 |
| mg | 2 | *6.4* | 358 | 353 | *6.3* | 405 | 409 | *4.8* | 260 | 260 | *4.8* | 316 | 312 |
|  | 3 | *7.4* | 354 | 355 | *7.3* | 398 | 401 | *5.7* | 256 | 255 | *5.5* | 299 | 299 |
|  | 4 | *14.5* | 342 | 350 | *10.7* | 401 | 410 | *9.4* | 254 | 250 | *10.1* | 301 | 302 |
| **Linear trend, *p*** |  |  | **0.25** | **0.56** |  | **0.72** | **0.50** |  | **0.89** | **0.25** |  | **0.86** | **0.88** |
| Calcium, | 1 | *603* | 355 | 357 | *584* | 411 | 412 | *544* | 257 | 255 | *530* | 323 | 324 |
| mg | 2 | *790* | 354 | 354 | *752* | 406 | 405 | *705* | 258 | 258 | *687* | 300 | 307 |
|  | 3 | *998* | 349 | 349 | *1021* | 400 | 401 | *866* | 252 | 254 | *858* | 296 | 294 |
|  | 4 | *2319* | 344 | 342 | *1940* | 392 | 390 | *1895* | 257 | 256 | *1918* | 295 | 287 |
| **Linear trend, *p*** |  |  | **0.058** | **0.017** |  | **0.24** | **0.26** |  | **0.75** | **0.92** |  | **0.006** | **0.001** |
| Iron, | 1 | *11.2* | 353 | 354 | *10.9* | 418 | 417 | *8.1* | 258 | 260 | *7.8* | 304 | 304 |
| mg | 2 | *13.3* | 354 | 354 | *12.7* | 390 | 395 | *9.7* | 254 | 253 | *9.4* | 313 | 312 |
|  | 3 | *15.4* | 347 | 349 | *14.8* | 404 | 396 | *11.6* | 257 | 257 | *11.3* | 296 | 300 |
|  | 4 | *26.5* | 347 | 345 | *22.6* | 396 | 399 | *23.1* | 254 | 252 | *21.1* | 301 | 297 |
| **Linear trend, *p*** |  |  | **0.16** | **0.27** |  | **0.31** | **0.48** |  | **0.56** | **0.37** |  | **0.41** | **0.51** |
| Vitamin B1, | 1 | *1.45* | 346 | 345 | *1.48* | 397 | 387 | *1.01* | 256 | 260 | *1.02* | 305 | 298 |
| mg | 2 | *1.70* | 355 | 351 | *1.70* | 410 | 408 | *1.22* | 256 | 257 | *1.22* | 299 | 299 |
|  | 3 | *1.94* | 353 | 356 | *1.93* | 392 | 392 | *1.43* | 259 | 257 | *1.43* | 305 | 305 |
|  | 4 | *3.06* | 347 | 350 | *3.09* | 408 | 419 | *2.20* | 251 | 248 | *2.38* | 306 | 311 |
| **Linear trend, *p*** |  |  | **0.98** | **0.54** |  | **0.77** | **0.33** |  | **0.47** | **0.15** |  | **0.83** | **0.36** |
| Vitamin B2, | 1 | *1.24* | 354 | 358 | *1.25* | 404 | 411 | *1.00* | 257 | 259 | *1.00* | 319 | 326 |
| mg | 2 | *1.55* | 352 | 351 | *1.53* | 400 | 402 | *1.25* | 256 | 256 | *1.24* | 298 | 300 |
|  | 3 | *1.88* | 348 | 347 | *1.89* | 418 | 414 | *1.53* | 253 | 253 | *1.49* | 301 | 300 |
|  | 4 | *3.95* | 348 | 345 | *3.37* | 388 | 382 | *2.97* | 257 | 255 | *3.20* | 296 | 287 |
| **Linear trend, *p*** |  |  | **0.28** | **0.07** |  | **0.56** | **0.26** |  | **0.74** | **0.54** |  | **0.031** | **0.004** |
| Vitamin B6, | 1 | *1.61* | 345 | 342 | *1.71* | 403 | 400 | *0.98* | 253 | 255 | *0.99* | 297 | 299 |
| mg | 2 | *1.89* | 352 | 350 | *1.94* | 398 | 402 | *1.20* | 256 | 258 | *1.22* | 310 | 304 |
|  | 3 | *2.16* | 352 | 355 | *2.22* | 401 | 400 | *1.45* | 259 | 257 | *1.49* | 307 | 308 |
|  | 4 | *3.53* | 352 | 355 | *3.86* | 406 | 406 | *2.34* | 255 | 252 | *2.55* | 300 | 302 |
| **Linear trend, *p*** |  |  | **0.30** | **0.08** |  | **0.86** | **0.84** |  | **0.54** | **0.65** |  | **0.86** | **0.72** |
| Vitamin B12, | 1 | *4.49* | 345 | 348 | *4.64* | 408 | 409 | *3.23* | 257 | 257 | *3.38* | 301 | 303 |
| mg | 2 | *5.67* | 355 | 353 | *6.02* | 401 | 399 | *4.13* | 253 | 253 | *4.41* | 295 | 295 |
|  | 3 | *7.16* | 350 | 350 | *7.59* | 391 | 391 | *5.43* | 253 | 254 | *5.62* | 306 | 304 |
|  | 4 | *16.59* | 351 | 351 | *19.56* | 408 | 409 | *15.43* | 260 | 258 | *11.82* | 312 | 312 |
| **Linear trend, *p*** |  |  | **0.60** | **0.78** |  | **0.86** | **0.88** |  | **0.55** | **0.71** |  | **0.16** | **0.27** |
| Folate, | 1 | *175* | 349 | 349 | *181* | 406 | 399 | *129* | 253 | 255 | *129* | 310 | 309 |
| mg | 2 | *208* | 354 | 351 | *210* | 389 | 395 | *158* | 254 | 254 | *158* | 295 | 296 |
|  | 3 | *243* | 349 | 350 | *243* | 396 | 393 | *191* | 260 | 259 | *192* | 312 | 307 |
|  | 4 | *423* | 349 | 351 | *391* | 417 | 422 | *324* | 256 | 253 | *345* | 298 | 301 |
| **Linear trend, *p*** |  |  | **0.77** | **0.86** |  | **0.43** | **0.30** |  | **0.37** | **0.96** |  | **0.55** | **0.73** |

^1^ Q, quartile of intake, ^2^ Unadj, unadjasted values, ^3^ adj, multiple adjustment for age decade, BMI, GFR-SKD-EPI, dietary energy intake kJ/day (except for Energy quartiles), use of diuretics, anti-gout medication, alcohol intake >10g/day, diabetes, hypertension, physical activity
